# Clinical application of digital technology in the reconstruction of soft tissue defects of the lower extremity with free superficial circumflex iliac artery flap

**DOI:** 10.3389/fsurg.2022.956800

**Published:** 2022-09-02

**Authors:** Jiayu Li, Xuchao Luo, Anming Liu, Yonggen Zou

**Affiliations:** ^1^ Department of Orthopedics, The Affiliated Traditional Chinese Medicine Hospital of Southwest Medical University, LuZhou, China; ^2^ Department of Orthopedics, GuiZhou Provincial People's Hospital, GuiYang, China

**Keywords:** digital technology, superficial circumflex iliac artery flap, lower extremity defect, reconstruction, microsurgery

## Abstract

**Objective:**

This paper aims to investigate the feasibility and clinical effectiveness of digital technology in the clinical application of free superficial circumflex iliac artery flap (SCIP) for repairing soft-tissue defects in the lower extremities.

**Methods:**

From January 2020 to December 2021, 16 patients with lower extremity soft tissue defects requiring flap repair were selected, and preoperative 3D digital reconstruction of the abdominal donor area and lower extremity recipient area were performed using digital technology combined with highly selective abdominal computed tomography angiography, and virtual design and flap cutting were performed using the software self-contained tool. During the actual surgery, the intraoperative design and excision of the superficial iliac circumflex artery were guided by the preoperative digital design, and the donor sites of the flap were closed directly.

**Results:**

In all cases, digital models of the donor area of the abdominal SCIP were successfully established, which could clearly showed the distribution, course, and diameter of the main trunk and the perforators and other relevant anatomical information and successfully guided the design and excision of the flap during surgery. All flaps successfully survived after surgery, and both the flap recipient and donor sites healed in one stage. All patients were followed up for 2–12 months on average (mean 8.6 months), and the flaps were not bulky and had a satisfactory appearance, with no significant difference in color compared with the surrounding skin and a little pigmentation around the flap. Only linear scarring was left in the donor areas, and there was no restriction of hip movement.

**Conclusion:**

This study used digital technology combined with a SCIP to repair lower extremity soft-tissue defects. The preoperative three-dimensional reconstruction of the digital model of the flap optimally designed the surgical plan, reduced the surgical risk and difficulty, shortened the surgical time, and had some significance for clinical precision, safety, and personalized design of the abdominal flap.

## Introduction

Soft tissue defects of the lower extremity have always been a major problem for reconstructive surgeons because of the large defect area, irregular trauma, and various tissue types (1). Since its application, the abdominal flap has gradually replaced the anterolateral thigh perforator flap (ALTP) as the flap of choice for repairing extremity trauma because of its large resectable area, rich blood supply selection, variety of portable tissues, and hidden donor site (2). Except for these points, in our experience (3), because of its advantages such as no need for second-stage surgical thinning and muscular dissection, and the ability to primarily close the complex donor area, the lower abdomen provides a desirable tissue reserve for covering the limb lesions where the hairless and thinned skin are required, and it is better suited to repair lower extremity defects than ALTP. Iida et al. (4) even reported a case in which a superficial circumflex iliac artery flap (SCIP) flap was applied to cover a defect on the ankle in a 1-year-old child. However, the variability of abdominal vessels is high, and the use of the handheld Doppler ultrasound to localize the vessels preoperatively also has a high rate of false positives and false negatives (5), which makes preoperative localization of vessels more difficult, and the current open-observation-operation surgical approach is more damaging to the donor area, thus limiting the clinical application of abdominal flaps. Digitization technology is an important research direction in computer image processing in recent years and has been widely used in the study of human blood vessels and bones (6, 7). Feng et al. (8) published in 2017 that computed tomography angiography (CTA) and color doppler ultrasound preoperative vessel imaging navigation can optimize the surgical strategy preoperatively. So, to achieve accurate preoperative localization of the perforators, acquisition of flap vascular anatomical information, safe range of blood supply, precise design of flaps, and reduce the risk of surgery, we have incorporated digital technology into the application of the free superficial circumflex iliac artery flap for the reconstruction of soft-tissue defect of the lower extremity. From January 2020 to December 2021, we used the free SCIP to repair soft-tissue defects in the lower extremity in 16 cases, and all of them used digital technology combined with highly selective abdominal computed tomography angiography (catheter-based CTA) (9) to perform 3D digital reconstruction of the abdominal donor area and lower extremity recipient area before surgery to clarify the anatomical information and vascularity of the donor–recipient site, and the flaps were designed according to the recipient area, which is reconstructed on the computer, and all of them acquired satisfactory results. The purpose of this study was to review the use of digital technology in repairing soft tissue defects of the lower extremity with free SCIP.

## Patients and methods

From January 2020 to December 2021, 16 patients with soft-tissue defects of the lower extremity underwent reconstruction surgery by the free SCIP using digital technology-assisted techniques. Patients who have diseases such as diabetes millitus, vascular diseases, heavy smoking histories, and injuries of the donor site, especially allergic to contrast media, were ruled out from the surgery. The age of patients ranged from 24 to 59 years (mean 44.4 years;10 males and 6 females). Among these patients, seven cases were caused by machine crush, four cases were caused by thermal injury, three cases were caused by traffic accident and other two cases sustained with empyrosis. The pain score (from 1 to 100 points) was used to assess the patients' recovery from pain. Also, the condition of scar growth was measured by the Vancouver scar scale (0–15 points). Detailed information about patients is summarized in Table 1. This study was approved by the ethical guidelines of the Hospital Ethical Committee of the Affiliated Traditional Chinese Medicine Hospital of Southwest Medical University. The protocol was performed in accordance with the ethical standards of the Helsinki Declaration of 1,975 and all subsequent revisions. All informed consent was verbally acquired from patients.

**Table 1:** Patients’ detail information.

Patient	Sex	Age (years)	Injured lower limbs	Cause of injury	Dimension of the defect (cm^2^)
1	M	24	L	Machine crush	12 × 7
2	F	36	R	Traffic accident	16 × 6
3	M	58	L	Thermal injury	11 × 4
4	M	51	R	Machine crush	4 × 9
5	M	32	L	Machine crush	9 × 8
6	M	53	L	Traffic accident	5 × 6
7	F	29	R	Thermal injury	5 × 10
8	F	55	R	Machine crush	11 × 3
9	M	54	L	Machine crush	9 × 5
10	F	43	R	Machine crush	6 × 7
11	M	44	R	Empyrosis	8 × 4
12	M	38	L	Thermal injury	11 × 6
13	M	37	L	Traffic accident	14 × 8
14	F	56	L	Empyrosis	16 × 4
15	M	42	L	Thermal injury	9 × 12
16	F	59	R	Machine crush	8 × 7
Average		44.44			
STDEV		13.13			

M, male; F, female; L, left; R, right. Male: 62.5%, female: 37.5%.

## Technique

After admission, the patients all underwent emergency debridement, the wound was flushed with a large amount of saline and hydrogen peroxide solution, the wound contamination was thoroughly removed, necrotic muscles and tendons were cut out, the bone defects were filled with antibiotic bone cement, and the wound was flushed again with saline and hydrogen peroxide solution until it was clean inside. The wound was covered with a vacuum sealing drainage (VSD) device after completion of the debridement. After the wound was clean, the infection was controlled, the bacterial culture of the exudate indicated no bacterial culture, and the surgery was scheduled. Joint dislocations and fractures were fixed with K-wires or external fixation braces.

## Digital reconstruction

Preoperative plain CT scans of the lower extremities and highly selective superficial iliac circumflex artery (SCIA) CTA were recorded, and all patients underwent an iodine allergy test before abdominal CTA. Digital subtraction angiography (DSA) was first used to perform a superficial iliac circumflex artery angiogram in the donor area, and a catheter was placed at the beginning of the artery. The contrast media was injected at a rate of 3 ml/s, with automatic monitoring and manual triggering. CTA scan parameters were set at 120 kV, 200 mAs, and 5 mm layer thickness (which could finally be disassembled into 0.625-mm-thick original images). A 128-row spiral CT was used for continuous scanning, and the scan area was set from the whole abdominal conventional scan area to the root of the thigh. All areas of the patient that do not need to be scanned were properly protected by lead clothing. The resulting raw CTA scan images were imported into Mimics 20.0 software (Materialise, Belgian workstation) in the DICOM format. Extraction and 3D reconstruction of blood vessels and soft tissues were performed using the software's tools according to the different gray values of the tissues. The anatomical information of the SCIA in the abdomen was detected, and a 3D visualized digital model of the donor site including blood vessels, soft tissues, and skin was established. The plain CT scan images of the lower limbs were imported into Mimics 20.0 software in the DICOM format to reconstruct a three-dimensional visualization model of both lower limbs, and the soft tissue defect model was obtained on the affected side by using the software's tools to compare and compute the mirror image of the affected side and the healthy side. Then, the flap shape was adjusted according to the requirements of defect repair with an area enlargement of 5%–10% (10), and the soft tissue defect model was finally obtained.

Using the resulting three-dimensional visualization digital model of the donor–recipient area, the location of the perforators was precisely located, and the safe range of the blood supply to the perforators was defined. Based on the preoperative digital model of the soft-tissue defect in the recipient area and the condition of the donor–recipient area, the donor area was located at the center of the flap within the safe range of the blood supply to the perforators. The flap can be designed as a normal flap or as a complex one such as a lobulated flap or chimeric flap.

Using Mimics 20.0 software with “Cut” and other functions, we can integrate the information of the blood vessels and important surrounding anatomical tissues, simulate the resulting digital model of the SCIP, and repeat the virtual operation to develop the optimal surgical plan.

## Operation

The patient was positioned in the supine position, and after satisfactory general anesthesia, the location of the perforators was located and marked on the skin according to the preoperative design and anatomic landmark around the donor area, and the preoperative designed flap shape and vessels were outlined on the skin of the donor area. Because of the application of the novel method, all the flaps were harvested through the lateral approach (11) to the deep branch of the SCIA to secure the safe of the elevation of the flaps. The SCIA and accompanying veins and superficial epigastric veins were dissected out, the SCIAs were dissected to the beginning of the femoral artery or the beginning of the deep circumflex iliac artery, and the superficial epigastric veins were dissected to the beginning of the saphenous vein. All the locations of the perforator were found intraoperatively to be consistent with the preoperative design. The flaps were dissected according to the preoperative design. The flaps were thinned microscopically to a thickness of 0.6–1.0 cm, with an average of 0.8 cm. The lower limb defects were repeatedly flushed with a povidone iodine solution and saline, and the bone defects were repaired with antibiotic bone cement. The vessels of the recipient site were dissected microscopically; the dorsalis pedis artery and accompanying veins were dissected on the dorsalis pedis if the wound was in the ankle, and the anterior tibial artery and accompanying veins were dissected on the anterior tibia if the wound was located in the calf. The SCIA was anastomosed to the recipient artery; the SCIV is anastomosed to the accompanying vein of the corresponding artery, and the superficial epigastric veins were anastomosed to the proximal saphenous vein or small saphenous vein. The donor sites were closed directly in all cases.

## Result

Sixteen patients were successfully treated by digital SCIP flaps with a mean age of 44.44 ± 13.13 (range 24–59) years. Ten of the patients were male, and the remaining six patients were female (male: 62.5%, female: 37.5%). In all cases, repairment was performed at the second stage. All flaps survived completely, except for one case that had a postoperative venous crisis and survived after emergency surgical exploration to remove the stuck blood crust and release the vascular tip from compression. Through the postoperative summary, we found that the formation of a blood clot after anastomotic bleeding is the main cause of the compression of the vascular pedicle. All of the donor sites were healed well in one stage.

In total, the sizes of the flap ranged from 9×5 cm to 10×12 cm. The total surgery time varied from 4.0 to 8.5 h with an average time of 5.47 ± 1.4 h. The donor sites were closed primarily in all cases. The scar scoring was from 3 to 8, with an average of 4.6. The mean follow-up time was 9.88 months (ranging from 6 to 18) (Table 2). The flaps were not bulky and had a satisfactory appearance, with no significant difference in color compared with the surrounding skin, only a little pigmentation around the flap. Only linear scarring was left in the donor area, and there was no restriction of hip movement.

**Table 2:** Surgical outcomes.

Patients	Location of the flap	Area of the flap (cm^2^)	Operation time (hours)	Flap survival	Treatment of donor site	Pain score	Vancouver scar scale	Follow-up time (months)
1	L	13 × 8	4.5	Complete	P	45	3	10
2	L	18 × 8	5.5	Complete	P	10	6	8
3	R	13 × 5	4.0	Complete	P	0	3	12
4	R	5 × 10	3.5	Complete	P	55	8	8
5	R	10 × 9	6.0	Complete	P	20	4	6
6^a^	L	7 × 8	5.0	Complete^a^	P	40	5	6
7	R	6 × 12	5.5	Complete	P	45	3	12
8	R	12 × 4	4.5	Complete	P	30	4	6
9	L	11 × 6	6.0	Complete	P	45	4	12
10	L	8 × 8	4.0	Complete	P	40	4	18
11	L	9 × 5	5.0	Complete	P	40	7	10
12	R	12 × 7	6.5	Complete	P	50	5	12
13	L	15 × 9	4.5	Complete	P	60	7	6
14	R	17 × 5	7.0	Complete	P	35	3	12
15	R	10 × 12	8.5	Complete	P	10	3	10
16	L	9 × 9	7.5	Complete	P	0	5	10
Average			5.47			32.81	4.63	9.88
STDEV			1.4			19.15	1.58	

L, left; R, right; P, primary closure.

^a^ The flap had a postoperative venous crisis and survived after emergency surgical exploration to remove the venous thrombus and release the vascular tip from compression.

## Case report

### Case 3

**Figure 1:**
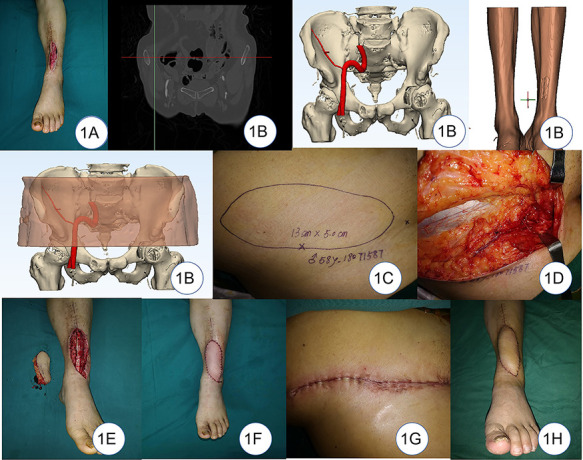
(**A**) Soft-tissue defect on the left leg. (**B**) Digital reconstruction of donor and recipient sites. (**C**) Design of the flap. (**D**) Location of the vessels seen intraoperatively was found to be generally consistent with the preoperative simulation design. (**E**) Flap and the defect. (**F**) Coverage of the defect. (**G**) Primary closure of the donor site. (**H**) 12-month follow-up after the operation.

A 58-year-old male was injured by a thermal injury that caused a soft-tissue defect on the left anterior tibial. The wound was covered with a vacuum sealing drainage (VSD) device after the completion of emergency debridement. The flap coverage was performed at the second stage (Figure 1A). The SCIA vasculature and its perforators were localized by the highly selective CTA scan preoperatively and reconstructed on the computer. A plain CT was used to scan the lower limb, and the data were transferred to the software to reconstruct the recipient site. The digital and virtual flap design, excision, and repairment were successfully completed preoperatively (Figure 1B). The location and course of the vessels seen intraoperatively were generally found consistent with the preoperative simulation design (Figures 1C,D). The size of the defect was 11×4 cm, so the size of the flap was 13×5 cm based on the surgical protocol (Figure 1E). The artery and vein of the flap were anastomosed to the anterior tibial artery and vein in the recipient site, respectively (Figure 1F). The donor site was closed directly (Figure 1G). The postoperative course was uneventful 7 days after the operation. The patient was followed 12 months after the operation (Figure 1H), the appearance of the flaps showed satisfactory contour, and there was no excessive bulk and no significant difference in color compared with the surrounding skin. The pain score was 0. The scar score was 3 points. The donor site healed well in one stage, and there was no restriction of hip movement.

## Discussion

In this study, precise coverage, esthetic contour, higher operation safety, and minimal donor-site morbidity can be achieved by combining digital technology and SCIP flap. It avoids the risks associated with vascular variants, shortens the operation time, and makes the preoperative vessel navigation accurately. Moreover, the three-dimensional model can be used to practice surgical skills and learn about the anatomy of SCIP for young plastic surgeons repeatedly.

With the development of digital imaging technology and the improvement of computer processing capability, digital technology has begun to be gradually applied to various clinical specialties in the medical field. For flap microsurgery, Cohen et al. (12) found that the ALT flap cutaneous perforator can be reconstructed on a computer based on the CTA scan images using software such as Amira and Mimics. The reconstruction accuracy of anatomical information of the lateral femoral circumflex artery vasculature was high, and the perforators could be reconstructed clearly. Furthermore, Eder et al. (13) used the preoperative CTA to predict the volume in autologous breast reconstruction and design the abdominal flap and achieved an acceptable symmetry. Pereira et al. (14) found a new planning method to harvest the SCIP with CTA, and it was regarded as a reliable technique for preoperative planning, allowing a safe and predictable elevation of the flap. However, there are shortcomings of traditional CTA, such as unsatisfactory visualization of small perforators (15), a lack of clarity in imaging, a lack of simplicity in locating perforators (16), and difficulty in designing and simulating the surgery (17). Based on these points, Tang et al. (18) proposed DSA combined with CTA, which was successfully applied to the design and application of the descending branch of the lateral femoral circumflex artery double-skin paddle flap, making the application of digital technology in flap surgery more precise and individualized. In this study, based on the original images of highly selective CTA scans, we used Mimics software to perform 3D digital reconstruction of the SCIA and abdominal soft tissues, used the software's functions to locate the flap vessels, design the flap, and simulate the cutting, and obtained a clear outline of the model image, which can accurately and truly reflect the anatomical information of the SCIA and its perforators.

The donor area of the SCIA can not only be dissected with a free flap but can also be used to cut multitissue inset flaps, lobulated flaps, lymphatic flaps, etc. (19, 20) based on the superficial branch of the extraabdominal oblique tendon branch, lymph node branch, deep branch of the suture muscle branch, and iliac branch, so they are more commonly used in abdominal flaps. However, there are disadvantages such as high vascular anatomical variability in diameter and alignment, which makes surgery difficult. The complex shape of the lower extremity trauma and the variety of tissue defects, often including skin, fascia, tendon, and even bone defects, make the design of the donor area complicated (21). Based on our perspective, all the complications mentioned above can be solved by digital reconstruction. The CTA scan can obtain images of all tissues of the donor site, which can be used to reconstruct the digital model on the computer by software. Then, a simulation of the operation can be performed to harvest the flap with other needed tissue precisely according to the defect. Regarding the elevation of these kinds of the flap, Yoshimatsu et al. (22) introduced a modified technique to harvest the SCIP flap proximal-to-distally to acquire a flap with a variety of anatomical structures. The deep branch of the SCIA should be included when anatomical structures perfused by the deep branch were procured, and the use of the transverse branch of the deep branch of the SCIA as the landmark for identification and dissection of the deep branch of the SCIA was considered a safe method by Yoshimatsu et al. (23).

In our experience of this technique, the advantages are as follows: (1) This approach changed the traditional operation method from open-observation-operation to observation-open-operation, prevented greater trauma to the patient, shortened the operation time, and reduced surgical blindness. (2) The anatomical information of the flap such as the distribution, course, and length of the vessel tip can be shown more clearly and completely. Due to the use of a highly selective CTA scan and directed placement at the arterial opening, it is possible to see the finer perforators and clarify the safe range of the flap blood supply, preventing surgical risks. (3) The flaps were designed according to the reconstruction requirements of the recipient area preoperatively and could even complete the design of lobed flaps, chimeric flaps, oversized flaps, and composite tissue flaps. Moreover, key information such as flap size, shape, and spatial relationship with surrounding vessels was clarified preoperatively, enabling the precise and individualized design of the SCIP. (4) The flap could be simulated before surgery, and the simulation was repeatable. Multiple simulations were possible, so that the surgeon could repeat the simulated operation to develop the most optimal surgical plan. (5) By simulating the intraoperative flap structure anatomy through preoperative 3D digital reconstruction, the combination of anatomy and clinic was truly achieved, providing real materials for clinical teaching of microsurgery, thus improving the clinical teaching content, especially in the current situation of shortage of anatomical materials, and this program could well make up for the shortage of teaching.

The main drawbacks of this technique are as follows: (1) The CTA scan cannot visualize the venous vessels, so the digital technique also cannot reconstruct the venous vessels and provide a clear preoperative view of the venous flap system. (2) Due to the use of highly selective CTA scans, which require advanced directional placement of the catheter at the artery, patients are exposed to higher radiation doses than plain CTA. (3) Highly selective CTA uses directional placement of the catheter at the opening of the artery, and injection of the contrast media during CTA can cause pressure dilation of the vessel, resulting in inconsistent preoperative and intraoperative measurements. (4) During the operation, it is necessary to cut out the flap pattern according to the preoperative design by the surgeon, which cannot be completely “precise,” but the combination of 3D printing technology can print out the preoperative design of the flap shape and complete the “cloth pattern” production in advance to guide the intraoperative flap dissection, and we are working on this issue.

Several tips for this approach can be shared, as follows: (1) Because highly selective CTA requires advanced DSA directional placement, it is an invasive operation, and the patient's age should be selected between 20 and 60 years. All patients are required to undergo a contrast media allergy test before imaging. (2) Since this technique cannot reconstruct the venous flap system, care should be taken to prevent injury to the SCIV when dissecting the SCIA and the superficial abdominal venous traffic branches. (3) The diameter of the SCIA is highly variable, and because of the phenomenon of pressure dilatation of the vessel, the preoperative measurements are slightly larger than the intraoperative measurements and often do not match the diameter of the recipient's vessel. When there is a mismatch, it is advisable to use end-lateral anastomosis, which requires a high level of microscopic anastomosis technique for the surgeon. (4) To avoid the occurrence of compression on the vascular pedicle, the quality of the vascular anastomosis is very important. Therefore, we recommend that the anastomosis should be performed by a highly qualified plastic surgeon.

## Conclusion

Our experience in this study showed that the application of the digital technology with SCIP to repair lower extremity soft-tissue defect can optimize the design of the surgical plan, reduce the surgical risk and difficulty, shorten the surgical time, and has some significance for clinical precision, safety, and personalized design of the abdominal flap.

## Data Availability

The original contributions presented in the study are included in the article/Sup**p**lementary Material, further inquiries can be directed to the corresponding author/s.
